# Receptor activator of nuclear factor-κB ligand-mediated osteoclastogenesis signaling pathway and related therapeutic natural compounds

**DOI:** 10.3389/fphar.2022.1043975

**Published:** 2022-11-09

**Authors:** Zechao Qu, Bo Zhang, Lingbo Kong, Yining Gong, Mingzhe Feng, Xiangcheng Gao, Dong Wang, Liang Yan

**Affiliations:** Department of Spinal Surgery, Honghui Hospital of Xi’an Jiaotong University, Xi’an, China

**Keywords:** osteoporosis, osteoclastogenesis, RANKL, signaling pathway, natural compounds

## Abstract

Osteoclast is a hematopoietic precursor cell derived from the mononuclear macrophage cell line, which is the only cell with bone resorption function. Its abnormal activation can cause serious osteolysis related diseases such as rheumatoid arthritis, Paget’s disease and osteoporosis. In recent years, the adverse effects caused by anabolic anti-osteolytic drugs have increased the interest of researchers in the potential therapeutic and preventive effects of natural plant derivatives and natural compounds against osteolytic diseases caused by osteoclasts. Natural plant derivatives and natural compounds have become major research hotspots for the treatment of osteolysis-related diseases due to their good safety profile and ability to improve bone. This paper provides an overview of recent advances in the molecular mechanisms of RANKL and downstream signaling pathways in osteoclast differentiation, and briefly outlines potential natural compounds with antiosteoclast activity and molecular mechanisms.

## Introduction

**FIGURE 1:**
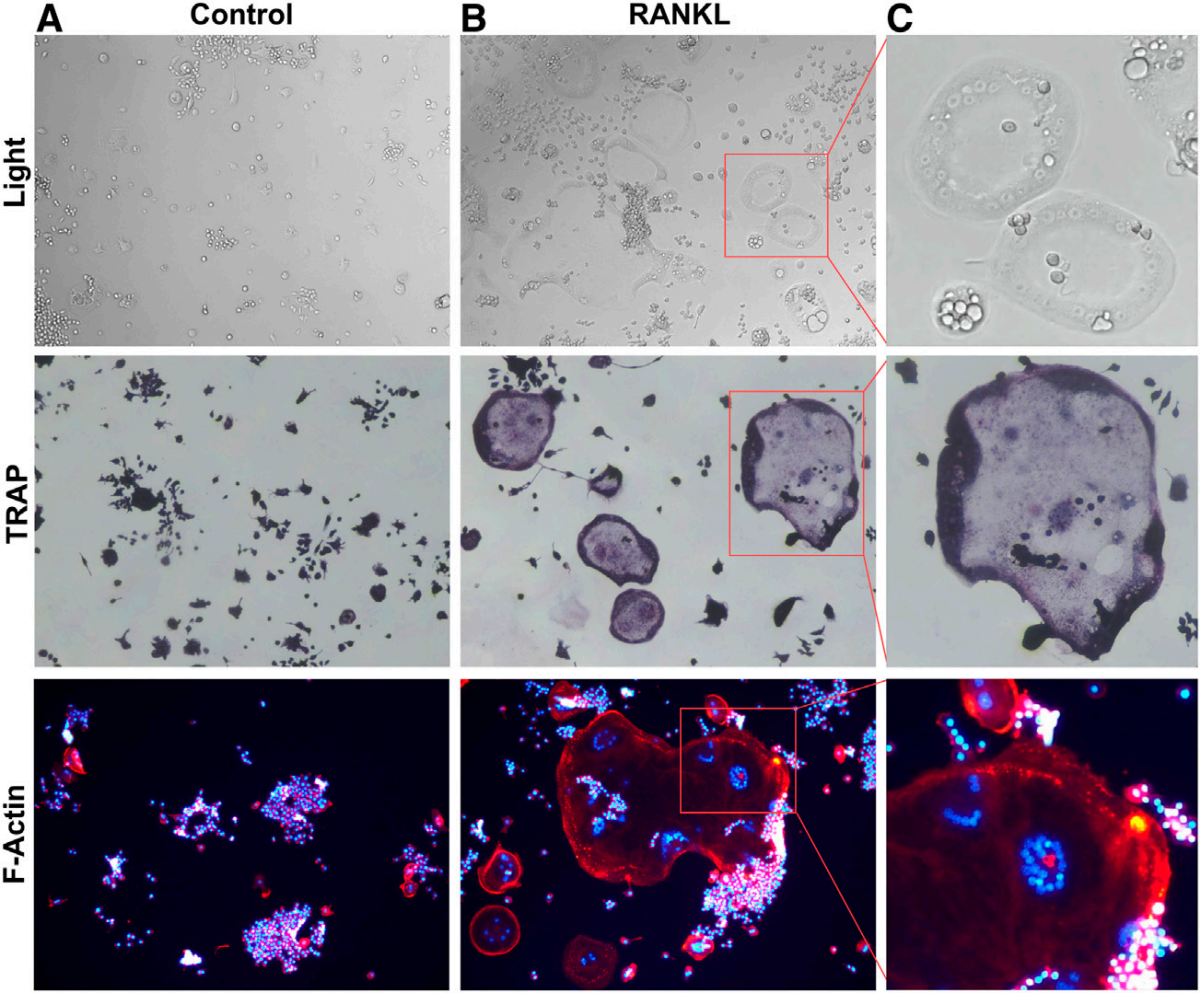
RANKL induced Osteoclastogenesis **(A)**. RAW264.7 cells were not stimulated by RANKL. **(B)**. RANKL-stimulated RAW264.7 cells differentiation into multinucleated osteoclasts. **(C)**. Box magnified of osteoclast.

Bone homeostasis is dynamically regulated by osteoclasts (OCs) mediated bone resorption and osteoblasts (OBs) mediated bone formation, contributing to a physiological balance of bone indexes and functions (An et al., 2016; Kim et al., 2020; Clézardin et al., 2021). Osteoporosis (OP) is the most common bone and a systemic metabolic bone disease characterized by reduced bone density and destruction of bone tissue microarchitecture, leading to increased bone fragility and susceptibility to fracture (Liel, 2018; Compston et al., 2019; Wang et al., 2022). The main causes of OP include endocrine factors, genetic and immunological factors, nutritional factors, gender and age factors, disease and medication factors, substance use and environmental factors (Rachner et al., 2011; Parveen et al., 2019). The abnormal differentiation of OCs is the critical pathological basis for OP (Curtis et al., 2016; Da et al., 2021). OCs are derived from a monocyte lineage of haematopoietic stem cell origin and are regulated by a variety of hormones and local cytokines, but receptor activator of nuclear factor-κB ligand (RANKL) and macrophage colony stimulating factor (M-CSF) are the main key molecules in the differentiation of OCs (Feng and Teitelbaum, 2013; Kodama and Kaito, 2020; McDonald et al., 2021). In the early stages, M-CSF plays a key role in the survival and proliferation of OCs precursor cells (Lee et al., 2018). The receptor activator of nuclear factor-κB (RANK) family of tumor necrosis factor (TNF) receptor proteins is expressed on the surface of OCs precursors, mature OCs and dendritic cells (Li et al., 2000). The crucial stage of their differentiation is the binding of RANKL produced by OBs to the RANK on OCs precursor cells to initiate intracellular downstream cascade transduction signals and the partial recruitment and activation of the bridging protein tumor necrosis factor receptor associated factor (TRAF6) in the cytoplasmic region of RANK. Then, activated TRAF6 further triggered the activation of downstream NF-κB, MAPKs, PI3K/AKT, calcium signaling and reactive oxygen pathways (Boyle et al., 2003; Feng and Teitelbaum, 2013; Hu et al., 2021; Udagawa et al., 2021). All of the above-mentioned genes are related to the formation and function of OCs (Takeshita et al., 2000). In summary, as shown in Figure 1, RANKL is an important regulator of the induced differentiation of OCs, and its related downstream signaling pathways have become potential targets for regulating the formation of OCs differentiation. The RANKL-RANK signaling pathway plays a critical role in the formation and function of OCs.

In the last 2 decades, there have been significant advances in drug treatments for OP, such as (BPs), estrogen therapy (ET), parathyroid hormone analogue (PTHa) and denosumab (Johnston and Dagar, 2020; Reid and Billington, 2022). In recent years, however, several studies have reported that some adverse effects associated with the long-term use of these drugs that may not be appropriate for oral medication. BPs are currently the first-line drugs for the clinical treatment of OP, while the overall evaluation is high, they are known to cause acute phase reactions, gastrointestinal discomfort, ocular discomfort (Favus, 2010; Oryan and Sahvieh, 2021). In 2003, it was first proposed that BPs could cause osteonecrosis of the jaw and significantly increase the incidence of esophageal cancer in patients (Wysowski, 2009; Verron and Bouler, 2014). ET has become a second-line option for the treatment of OP and is generally safe to use but may occur in estrogen-dependent tumours, thrombophilia, venous thromboembolism and fatal strokes (Rozenberg et al., 2020). PTHa plays a valuable role in promoting bone formation and is a current treatment for postmenopausal osteoporosis (Kraenzlin and Meier, 2011). Its adverse effects are nausea, limb pain, headache and dizziness, and can cause severe bone tumours (Ayub et al., 2021). Denosumab is the only antagonist approved to target RANKL (Bone et al., 2017). It inhibits the binding of RANKL to its receptor RANK and reduces the formation and activation of osteoclasts, thereby reducing bone resorption, increasing bone mineral density and reducing the incidence of fractures (Polyzos et al., 2019). However, it has been reported in the document that long-term use can cause hypercalcaemia and increase the risk of primary malignancies, as well as jaw osteonecrosis or atypical femur fractures and that abrupt discontinuation of the drug can cause an increased risk of multiple spinal fractures (Pang et al., 2020). Therefore, the search for highly effective and low-toxic anti-osteoporosis drugs is of clinical importance and medical value. In recent years, there has been an increasing number of studies on natural compounds that act mainly on pathways related to downstream of RANKL differentiation of OCs such as NF-κB, MAPKs, PI3K/AKT, calcium signaling and reactive oxygen species. Moreover, the inhibitory effect on OCs may also promote bone health through its anti-inflammatory and antioxidant properties. This essay aims to summarize the latest research progress of RANKL and its downstream signaling pathways in the differentiation of OCs and provide further insights into the use of RANKL as a drug target for the treatment of OP. In addition, we briefly described several natural plant derivatives and natural compounds that inhibit the differentiation of OCs and their preventive effects on osteolysis diseases by modulating RANKL-related signaling pathways.

## Receptor activator of nuclear factor-κB ligand-mediated classical signaling pathways

### Receptor activator of nuclear factor-κB ligand/NF-κB signaling pathway

In the early stages of the downstream signaling pathway activated by RANKL, the activation of NF-κB plays a critical regulatory role in the formation of OCs (Abu-Amer, 2013). Jimi et al. (2019) showed that NF-κB knockout mouse models exhibited severe osteosclerosis due to impaired OCs formation, which demonstrated that activation of NF-κB is a cytokine necessary for OCs formation. NF-κB is a family of dimeric transcription factors with five members: RelA (p65), RelB, Rel (c-Rel), NF-κB1 (p50) and NF-κB2 (p52) (Boyce et al., 2010). In the resting state, NF-κB is present in the cytoplasm as a heterodimer of two subunits of p50/p65 and binds to the inhibitor IκB to form a complex (Novack, 2011). Phosphorylation of IκB is a prerequisite for NF-κB cascade activation, which is regulated by the IκB kinase (IKKs) complex, it phosphorylates IκB, degrades the IκB proteasome, translocates the active NF-κB dimer released from IκB into the nucleus, and causes transcription and expression of specific genes for OCs differentiation (Hirata et al., 2019).

### Receptor activator of nuclear factor-κB ligand/MAPK signaling pathway

Mitogen-activated protein kinases (MAPKs) signaling pathway are also involved in various cellular activities such as gene expression, mitosis, differentiation, proliferation and transformation (Oikawa et al., 2007; Lee et al., 2018). MAPKs is a member of the second messenger family that transmits cell surface signals to the nucleus in response to a variety of hormones, chemicals and external stimuli (Zhang et al., 2021). MAPKs are composed of three main kinases: extracellular signal-regulated kinase 1/2 (ERK1/2), Jun N-terminal kinase (JNK) and p38-MAPK, all involved in the formation of OCs and can be activated by RANKL stimulation (Lee et al., 2016). During the formation of OCs differentiation, RANKL binds to the receptor RANK on OCs precursors to activate MAPKs signaling molecules, thus further inducing the expression of transcription factors such as NFATc1 and AP-1 to promote OCs differentiation (Liao et al., 2021). The p38-MAPK signaling pathway is particularly significant in the early stages of OCs differentiation, promoting the expression of Micropthalmia-associated transcription factor (MITF) and TRAP, which ultimately promote OCs differentiation (Lu et al., 2017). ERK1/2 and JNK also play a crucial role in the formation of OCs and have been shown to promote the differentiation of OCs by upregulating the expression of c-Fos and c-Jun (Xiao et al., 2020).

### c-Src/PI3K/Akt signaling pathway

PI3K/Akt signaling pathway was involved in pathological bone diseases such as osteoporosis, osteoarthritis and osteosarcoma, and in regulating the proliferation, differentiation and apoptosis of OCs and OBs (Hinz and Jücker, 2021). The RANK-TRAF6 complex may induce activation of the PI3K/Akt signaling cascade by recruiting the Src kinase family. The c-Src kinase, a member of the Src family, is essential for the differentiated formation of OCs and the uptake function of mature OCs (Kong et al., 2020). c-Src stimulates phosphatidyl inositol 3-kinase (PI3K) and activates PI3K I phosphorylate phosphatidylinositol 4,5-bisphosphate (PIP3) (Fattahi et al., 2020). PIP3 is a “second messenger” used by many cell surface receptors to control mitosis, growth, survival and differentiation, and it can recruit serine/threonine kinase (Akt) expression to the plasma membrane (Wang et al., 2021). PI3K/Akt signaling additionally enables the binding of Smad1/5 and CBP (CREB binding protein) to regulate M-CSF expression in osteoblasts, thereby promoting the differentiation of OCs (Miyazaki et al., 2004; Mandal et al., 2009). PI3K stimulates the formation of actin filaments and regulates cytoskeletal functions such as chemotaxis, adhesion and spreading (Irelli et al., 2019). It has been indicated that inhibition of PI3K can block the bone resorption function of mature osteoclasts (Nakamura et al., 1995; Smith et al., 2007). Moon et al. (2012) found that overexpression of Akt promoted the formation of the inactive form of GSK3β and nuclear localization of NFATc1, while overexpression of the active form of GSK3β reduced osteoclast formation by downregulating NFATc1. This suggests that Akt could induce osteoclastogenesis by activating the GSK3β/NFATc1 signaling axis. Kawamura et al. (2007) showed that Akt1 is a key regulator of OBs and OCs, maintaining bone mass and bone transformation by promoting osteoblast and osteoclast differentiation and survival. PI3K/Akt signaling plays an essential role in the survival of OCs. However, its specific mechanism of action still needs to be further explored.

### Ca^2+^/calcineurin/NFATc1 signaling pathway

The Ca^2+^ signal is important in osteoclastogenesis for various functions, including cell proliferation, differentiation, gene transcription, bone resorption and so on (Kang et al., 2020). RANKL-induced calcium oscillations are closely related to osteoclast differentiation, bone matrix resorption and apoptosis of OCs (Okada et al., 2020). Ca^2+^ is released from the endoplasmic reticulum or enters the cell through plasma membrane ion channels to generate Ca^2+^ oscillations, which stimulate calcium-regulated phosphatase and cause dephosphorylation of intracellular NFATc1 and translocation into the nucleus, promoting differentiation of OCs (Crabtree and Schreiber, 2009; Hinz and Jücker, 2021). RANKL-mediated signaling is the first step in osteoclast differentiation. In the early stages of osteoclast formation, RANKL stimulates phospholipase Cγ (PLCγ), and activated PLCγ produces 1,4,5-trisphosphatidylinositol (IP3) in the cytoplasm (Lorenzo, 2017). IP3 directly increases the level of Ca^2+^ in cells by inducing the release of calcium from endoplasmic reticulum stores and Ca^2+^ influx through store-operated Ca^2+^ entry (SOCE) and transient receptor potential (TRP) channels, sustained calcium signaling upregulates target gene and expression of protein by inducing NFATc1 dephosphorylation and NFATc1 translocation to the nucleus, promoting osteoblast differentiation and formation (Kajiya, 2012; Erkhembaatar et al., 2017; Liu et al., 2020). Thus, long-term and stable Ca^2+^ oscillations are necessary to maintain NFATc1 concentration in the nucleus, ensure long-term transcriptional activation of NFATc1, and promote the formation of OCs.

### ROS-mediated effects

It has been shown that normal body metabolism can produce reactive oxygen species (ROS), either endogenously by nicotinamide adenine dinucleotide phosphate oxidase (NOX) or as a by-product of the mitochondrial electron transport chain (Ren et al., 2021; Zhu et al., 2022). ROS contains superoxide anion radicals (O_2_−), hydrogen peroxide (H_2_O_2_), hydroxyl radicals (-OH) and nitric oxide (NO) (Madreiter-Sokolowski et al., 2020). During aerobic respiration, these molecules, produced by the electron transport chain, can affect biological functions such as cell signaling and homeostasis (Cornelius et al., 2014). ROS are essential for the regulation of cell proliferation, survival, metabolism, apoptosis, differentiation and migration (Bacevic et al., 2017). It has been indicated that ROS is an important secondary messenger in cells, and under physiological conditions, ROS could regulate intracellular environmental homeostasis, signal transduction, proliferation and differentiation, apoptosis and other physiological activities, and was in dynamic balance with antioxidants (Agidigbi and Kim, 2019; Ni et al., 2020). ROS are molecules with dual roles. They are beneficial by acting as intracellular signaling factors and detrimental as increasing with age, inflammatory state or age-related diseases (Go and Jones, 2017). Excess ROS destroy bone by reducing the level of antioxidant enzymes and preventing the differentiation of OBs and the formation of OCs (Chen et al., 2021). During the differentiation in osteoclasts, RANKL binds to the receptor RANK, which activates TRAF6 to drive multiple downstream targets. NADPH oxidase 1 (NOX1) transfers electrons from NADPH to molecular oxygen to form ROS (Li et al., 2021b). ROS can also promote osteoblast differentiation and maturation by indirectly activating MAPK, PI3K and NF-κB activation and driving the expression of genes such as CTSK, MMP9, c-Fos, NFATc1 and so on (Morgan and Liu, 2011; Zhang et al., 2016; Bang et al., 2021). In addition, the Kelch-like ECH-associated protein1 (Keap1)/nuclear factor E2-related factor 2 (Nrf2)/antioxidant response element (ARE) signaling pathway is closely related to oxidative stress and is one of the antioxidant stress mechanisms in cells (Han et al., 2022). It is one of the intracellular antioxidant stress mechanisms, which can increase the levels of the antioxidant enzymes heme oxygenase-1 (HO-1), catalase (CAT), cysteine synthetase catalytic subunit (GCLC) and decreases intracellular ROS expression to inhibit osteoclast differentiation and resorption (DeNicola et al., 2011; Tu et al., 2019; Sánchez-De-Diego et al., 2021). Antioxidant therapy has been shown to be effective in rescuing bone loss induced by oxidative stress.

## Natural plant derivatives and natural compounds

Although many pharmacological products are available for the treatment of osteolytic diseases, in recent years it has been found that long-term use was often accompanied with serious adverse effects. Natural plant derivatives and compounds have anti-inflammatory and antioxidant properties that optimize bone, prompting researchers to focus on natural compounds to find effective drugs to inhibit OCs. As shown in Table 1, natural compounds inhibit OCs differentiation by regulating RANKL-mediated related pathways.

**TABLE 1:** The source, molecular structure, dose, cell lines and mechanism of 20 natural compounds on modulating osteoclat differentiation.

Compound name	Molecular structure	Source	Dose	*In vitro* studies cell lines	Mechanisms	Reference
Galangin	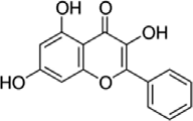	The rhizomes of *Alpinia officinarum*	0, 3, 6, 12 µM	BMMs	↓NF-κB and MAPKs signaling pathway;	Li et al. (2021a)
					↓(NFATc1, c-Jun and c-Fos)	
Biochanin A	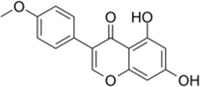	*Trifolium* pratense	0, 2, 4, 8, 16 µM	BMMs	↓NF-κB和/MAPKs signaling pathway;	Liao et al. (2021)
					↓(NFATc1, c-Fos ,IL-1αand IL-1β)	
Vinpocetine	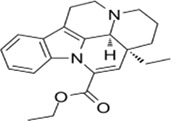	The alkaloid vincamine	0, 2.5, 5, 10, 20 µM	BMMs	↓NF-κB and MAPKs signaling pathway;	Zhu et al. (2020)
					↓(NFATc1, c-Fos ,TRAP and MMP-9)	
Robinin	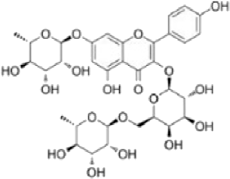	Vinca erecta Regel and Schmalh or Robinia pseudoacacia L	0, 0.25, 0.5, 1, 2,10, 20 µM	BMMs	↓NF-κB and MAPKs signaling pathway;	Hong et al. (2021)
					↓(Acp5, Cathepsin K, Atp6v0d2, Nfact1, c-Fos and Mmp9)	
Urolithin B	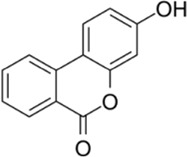	One of gut microbial metabolites of ellagitannins	0, 10, 30, 50, 100 µM	RAW264.7 cells	↓NF-κB and MAPKs signaling pathway;	Qu et al. (2022)
					↓(c-Fos, NFATc1, Cathepsin K, TRAP, OC-STAMP and Mmp9)	
Cinchonine	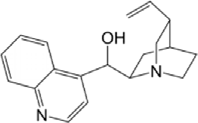	Cinchona bark	0, 10, 20, 30 µM	BMMs	↓AKT signaling pathway;	Jo et al. (2021)
					↓(NFATc1,AP-1, Dcstamp and Ctsk)	
Cnidium lactone	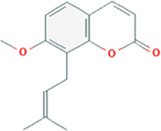	Cnidium monnieri	10^–6^, 10^–5^, 10^–4^ mol/L	RAW264.7 cells	↓PI3K-Akt signaling pathway;	Liang et al. (2019), Wang et al. (2020)
					↓(p38,Acp5,CtsK, Atp6v0d2,Tm7sf4,Oscar and Nfatc1)	
Anethole	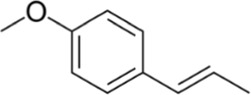	Active agent of more than 20 plants	0, 1.25, 2.5, 7.25, 10 µM	BMMs	↓PI3K-Akt signaling pathway;	Qu et al. (2021)
					↓(TRAP, NFATc1, V-ATPase d2, c-Fos, MMP-9, CTR, DC-STAMP, cathepsin K,)	
9,9′-O-di-(E)-fer-uloyl-meso-5,5′-55dimethoxysecoisolariciresinol	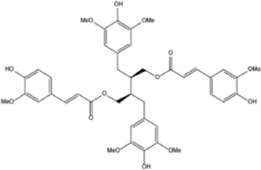	Litsea cubeba	0, 0.75, 1.5, 3, 15, 30 µM	BMMs	↓Akt signaling pathway ;	Yu et al. (2020)
					↓(NFATc1, c-Fos, MMP9)	
Acacetin	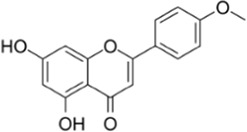	Damiana, Saussurea involucrata plant, and black locust plants	0, 1, 5, 10, 20, 50,100 µM	BMMs	↓Akt/GSK3βsignaling pathway;	Lin et al. (2022)
					↓Ctsk, MMP9, , DC-STAMP, OSCAR, c-Fos, NFATc1 NFκB and Atp6v0d2)	
Artesunate	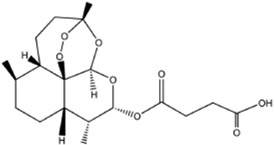	Artemisinin	0, 1.56, 3.125, 6.25, 12.5 µM	RAW264.7 cells	↓PLCγ1-Ca^2+^-NFATc1 signaling pathway;	Zeng et al. (2020)
					↓(Fra-2,TRAP, Cathepsin K, β3-integrin, DC-STAMP, and Atp6v0d2)	
Oleanolic acid acetate	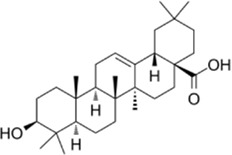	*Vigna angularis* (azuki bean)	0, 5, 10, 20 µM	BMMs,	↓PLCγ1-Ca^2+^-NFATc1 signaling pathway;	Kim et al. (2014)
				BMCs	↓(TRAP and OSCAR)	
Kirenol	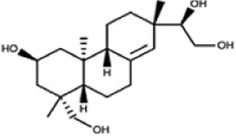	Herba Siegesbeckiae	0, 1.25, 2.5, 5, 10 µM	BMMs	↓Ca^2+^-NFATc1 signaling pathway;	Zou et al. (2021)
					↓(NF-κB p65、 p-p38、p-ERK and c-Fos)	
Asperpyrone A	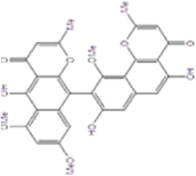	*Aspergillus niger*	0, 0.5, 1, 2.5, 5, 10 µM	BMMs	↓Ca^2+^ oscillation;	Chen et al. (2019)
					↓(TRAP, NFATc1, c-Fos, Ctsk and Atp6v0d2)	
Betulinic acid	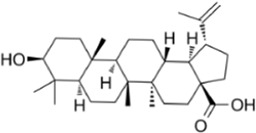	The bark of the birch tree, D. kaki leaf and so on	0, 1, 5, 10 µM	BMMs	↓PLCγ1-Ca^2+^-NFATc1 signaling pathway;	Jeong et al. (2020)
					↓(TRAP, OSCAR, NFATc1, c-Fos, MMP9,β3-integrin, Ctsk, Mafb, Bcl6, Blimp1 and Atp6v0d2)	
Loureirin B	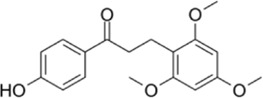	Sanguis draxonis	0, 1, 2.5, 5, 10 µM	BMMs	↓ROS activity,MAPK-NFAT signaling pathway;	Liu et al. (2019)
					↓(Acp5, Atp6v0d2, Ctsk, MMP9 and c-Fos)	
Schisandrin A	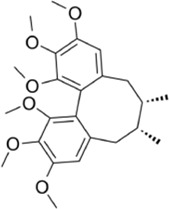	*Schisandra chinensis*	1, 50, 100, 200 µM	BMMs,	↓ROS activity;↑Nrf2 activity;	Ni et al. (2020)
				BMCs	↓(NFATc1,c-Fos,MMP9,TRAP)	
Oroxylin A	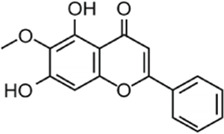	The root of *Scutellaria baicalensis* Georgi	0, 1, 2.5, 5, 10 µM	BMMs	↓ROS activity;↑Nrf2 activity;	Xian et al. (2021)
					↓(c-Fos,NFATc1, Ctsk, Atp6v0d2)	
Rhaponticin	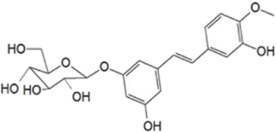	*Rheum undulatum* L	0, 6.25, 12.5, 25, 50 µM	BMMs	↓ROS activity; ↑CAT, SOD-2 and HO-1 activity	He et al. (2021)
					↓(c-Fos,NFATc1, Ctsk, Atp6v0d2,Hprt)	
Alpinetin	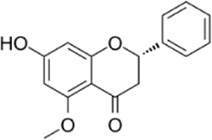	Alpinia Journal Pre-proof katsumadai Hayata	0, 5, 10, 15, 20, 30 µM	BMMs	↓ROS activity; ↑CAT, HO-1 and Nrf2 activity;	Wei et al. (2022)
					↓(c-Fos, Nfatc1, Ctsk, Tracp, Mmp9 and Dc-stamp)	

### Natural plant derivatives and compounds modulate the NF-κB and/or MAPKs pathway

Galangin, a natural bioflavonoid extracted from a traditional Chinese herb, has a variety of biological activities, including anti-inflammatory and antioxidant properties. (Li et al. (2021a) showed that during the differentiation of bone marrow macrophages (BMMs) to OCs, galangin inhibited the phosphorylation of p38 and ERK in the MAPK signaling pathway, as well as downstream factors such as NFATc1, c-Jun and c-Fos. It also inhibits bone resorption by inhibiting lipopolysaccharide (LPS)-induced differentiation of OCs. Their findings demonstrated that galangin can be a promising natural compound for the treatment of osteoporosis by inhibiting MAPK and NF-κB signaling pathways. Biochanin A (BCA) is one of the flavonoid compounds with a phenolic structure. Liao et al. (2021) demonstrated that BCA can effectively inhibit the production of OCs and the bone resorption of hydroxyapatite, and can downregulate the expression of NFATc1 and c-Fos by inhibiting MAPK and NF-κB pathways and inhibit the differentiation of OCs by reducing the expression of OCs-related genes. Vinpocetine (Vinp) is a derivative of the alkaloid vincristine. *In vitro* studies, Vinp significantly inhibited RANKL-induced differentiation of OCs and formation of F-actin, as well as reduced osteoclastic bone resorption function. In addition, Vinp reduced the activation of NF-κB, MAPK and AKT signaling pathways during osteoclast formation, blocked the production of ROS and inhibited the expression of the osteoclast-specific genes such as NFATc1, c-Fos, TRAP, MMP-9 and CTSK. The ovariectomized (OVX) rats model is a classic animal model for studying osteoporosis, which has the characteristics of single modeling factor, great repeatability, high reliability of experimental results, and can successfully simulate postmenopausal osteoporosis *in vivo*. *In vivo* studies have shown that Vinp significantly reduces the number of osteoclasts and attenuates ovariectomy-induced bone loss. Vinp could be a potential drug for the prevention and treatment of osteoporosis (Zhu et al., 2020). Robinin (Rob) is a flavonoid glycoside with anti-inflammatory and antioxidant effects. Hong et al. (2021) demonstrated that Rob could downregulate the expression and nuclear translocation of NFATc1 by inhibiting MAPK and NF-κB pathways during the differentiation of BMMs to osteoclasts and *in vivo* experiments demonstrated that Rob could prevent estrogen deficiency-induced bone loss. Urolithin B (UB), a polyphenolic compound, has been reported to possess various biological activities such as antioxidant and anti-inflammatory. Our previous study demonstrated for the first time that in RANKL-induced RAW264.7 cell differentiation, UB could inhibit NF-κB, MAPK and Akt pathways to reduce the expression of major transcription factors c-Fos and NFATc1, which could potentially prevent osteoclast-related bone disease (Qu et al., 2022).

### Natural plant derivatives and compounds modulate the Akt signaling pathway

Cinchonine (CN) is an alkaloid with anti-malarial, anti-platelet and anti-obesity effects. Jo et al. (2021) showed that CN inhibited RANKL-induced osteoclast differentiation and bone resorption. CN can inhibit the differentiation of OCs by suppressing TRAF6-mediated expression of TAK1 and AKT activity, resulting in downregulation of the key transcription factor NFATc1. In addition, CN attenuated LPS and ovariectomy-induced osteolysis in a mouse model of osteoporosis. This suggests that CN has therapeutic potential for the treatment of inflammation-induced bone disease and postmenopausal osteoporosis. Cnidium lactone is a potent herbal remedy that can prevent bone loss in a model of ovariectomy-induced bone loss in rats (Wang et al., 2020). Liang et al. (2019) found in a study of RANKL-induced differentiation of RAW264.7 cells that Cnidium lactone could inhibit the differentiation of TRAP-positive multinucleated osteoclasts and reduce the bone resorption of osteoclasts. In molecular mechanism, Cnidium lactone is able to inhibit the expression of c-Fos and NFATc1, the main transcription factors of osteoclast differentiation, by inhibiting the levels of phosphorylation of p38 and AKT and reducing the expression of p38 MAPK and PI3K-Akt signaling pathways. The results of the *in vitro* study suggest that cnidium lactone has the potential to be used as a new drug for the treatment of osteoporosis. Anethole, the main active ingredient in more than 20 plants, has anti-inflammatory, antioxidant, antibacterial, antifungal, anticancer and anesthetic properties. In addition, it has a potential function in protecting the kidneys and nerves. Qu et al. (2021) revealed anethole inhibited osteoclast differentiation and bone resorption by blocking ERK and AKT signaling pathways and reducing the expression of genes specific for osteoclast differentiation through *in vitro* and *in vivo* experiments. In an osteolysis model in ovariectomy-induced mice, anethole prevented bone loss and osteoclast activity induced by estrogen deficiency, suggesting that anethole has bone protection against osteoporosis. 9,9′-O-di-(E)-feruloyl-meso-5,5′-dimethoxysecoisolariciresinol (LCA) is an extract of Litsea cubeba with anti-inflammatory biological activity. Yu et al. (2020) showed that LCA could inhibit RANKL-induced AKT phosphorylation and the phosphorylation of JNK, ERK and p38 in the MAPK signaling pathway to downregulate the expression of NFATc1 and c-Fos, thereby inhibiting osteoblast differentiation. Acacetin is a natural flavonoid. Lin et al. (2022) found that acacetin inhibits osteoclast formation and activity by inhibiting Akt/GSK3β signaling pathway and phosphorylation of IκBα in NF-κB signaling pathway, and also induces H-type angiogenesis in OVX mice, which is important for maintaining normal bone structure.

### Natural plant derivatives and compounds modulate the Ca^2+^ signaling pathway

Artesunate, a semi-synthetic derivative of artemisinin, is widely used in the clinical treatment of falciparum malaria. Artesunate has anti-inflammatory and immunosuppressive properties and is indicated for the treatment of osteomyelitis, septic arthritis and periodontitis caused by gram-negative bacterial infections. In RAW264.7 cell culture and differentiation, Zeng et al. (2020) found that artesunate dose-dependently inhibited LPS-induced OCs formation and suppressed the expression of OCs differentiation related genes TRAP, Integrinβ3, MMP-9 and CTSK. Furthermore, artesunate significantly attenuated the expression of upstream TLR4/TRAF6 and downstream PLCγ1-Ca^2+^-NFATc1 signaling pathways in LPS-induced osteoclast differentiation, which reduced pathological activation of OCs and had potential therapeutic effects on bone erosion. Oleanolic acid acetate (OAA) is a triterpenoid compound isolated from Vigna angularis. LPS-induced inflammatory bone loss can serve as an animal model for *in vivo* osteolysis. LPS promotes the release of proinflammatory cytokines and ultimately induces osteoclastic bone erosion. Kim et al. (2014) indicated that OAA attenuated RANKL-induced osteoclastogenesis *in vitro* and suppressed LPS-induced bone loss *in vivo*. OAA significantly inhibited PLCγ2 phosphorylation, Ca^2+^ oscillation and NFATc1 expression in RANKL-stimulated bone marrow macrophages, however, did not affect RANKL-induced MAPKs expression. The results suggested that OAA inhibited RANKL-mediated osteoclastogenesis *via* the PLCγ2-Ca^2+^-NFATc1 signaling pathway and suppressed inflammatory bone loss *in vivo*. Therefore, OAA could be a potential drug for osteoclast-related diseases such as osteoporosis. Kirenol (Kir) is a bioactive diterpenoid with anti-rheumatic Chinese herbal medicine that promotes osteoblast differentiation *in vivo* for the treatment of arthritis. Zou et al. (2021) found that Kir significantly suppressed osteoclastogenesis and bone resorption *in vitro*. In mechanism, Kir remarkably inhibited the formation of actin rings, RANKL-induced activation of NF-κB p65, the expression of p-p38, p-ERK and c-Fos, while Kir also inhibited the expression of NFATc1 and nuclear translocation. Kir was found to reduce Ca^2+^ oscillations and caveolin-1(Cav-1) during osteoclast formation *in vitro*. In addition, Kir attenuated ovariectomy -induced osteoporosis by decreasing the number of osteoclasts *in vivo* studies by reducing the expression of Cav-1 and NFATc1. Asperpyrone A, a natural compound isolated from Aspergillus niger, has anti-tumor, antibacterial and antioxidant biological activities. Chen et al. (2019) found that Asperpyrone A inhibited RANKL-induced intracellular calcium oscillations and elevated ROS levels and suppressed MAPK and NF-κB signaling pathway activation, thereby downregulating the expression of NFATc1, c-fos to inhibit osteoclast formation and differentiation. Betulinic acid (BA) is a pentacyclic triterpene compound that is derived from natural plants and is known to possess many pharmacological and biochemical properties, including anti-inflammatory and anti-cancer activities. Jeong et al. (2020) found that BA inhibited RANKL-mediated intracellular levels of Ca^2+^ through inhibition of PLCγ2 phosphorylation and reduced OCs production and bone resorption through the combined effect of Akt and NF-κB phosphorylation, and meanwhile BA reversed inflammatory bone loss induced by LPS injection in mice.

### Natural compounds modulate ROS-mediated effects

Loureirin B (LrB) is the active ingredient isolated from Sanguis draxonis and is widely used in the treatment of stasis, oxidative stress, cancer, inflammation and immune disorders. Sanguis draxonis known as Dragon’s Blood, is a traditional Chinese herb containing more than 12 active compounds and has been used to treat diabetes and AIDS-related diarrhoea. Liu et al. (Liu et al., 2019) showed that LrB could inhibit osteoclast differentiation, bone resorption, actin ring formation and reduce osteoclast-specific gene expression, ROS activity and calcium oscillations by affecting NFATc1 translocation, expression and MAPK-NFAT signaling pathway *in vitro*. *In vivo* studies have shown that LrB may prevent ovariectomy-induced osteoporosis by inhibiting the activity and function of osteoclasts. Therefore, LrB is a potential drug for the treatment of osteoporosis. Schisanin A (Sch) is a dibenzocyclooctene lignan extracted from Schisan. It has been demonstrated to have anti-inflammatory, anti-coagulant, anti-depressant, anti-cancer, hepatoprotective and renal protective effects. Shuo et al. (Ni et al., 2020) showed that Sch could inhibit the differentiation of OCs and reduce the production of actin rings in OCs, thereby inhibiting their bone resorption function. In the mechanism, Sch further reduces ROS production during RANKL-induced osteoclastogenesis by downregulating the TRAF6/Nox1 signaling pathway and enhancing the expression of Nrf2. Sch attenuated ovariectomy-induced bone loss by acting on Nrf2 to inhibit *in vitro* production of ROS, as demonstrated in the vivo studies. *In vitro* and *in vivo* studies, it has been indicated that Sch is an antioxidant compound with anti-osteoporotic effects and that the Nrf2 signaling pathway could be a new target for the treatment of osteoclast-related diseases. Oroxylin A (OA) is an active flavonoid extracted from the roots of Scutellaria baicalensis Georgi, with a variety of biological activities including antioxidant, anti-apoptotic, anti-inflammatory and anti-tumor. Xian et al. (2021) showed that OA inhibited RANKL-induced osteoclast differentiation and reduced the bone resorption of hydroxyapatite by osteoclasts *in vitro* study. OA inhibited RANKL-induced ROS production by regulating the expression of various antioxidant enzymes such as CAT, HO-1 and GCLC, mediated by the transcription factor Nrf2. In addition, OA inhibited intracellular Ca^2+^ inward flow and downregulated the expression of the major transcription factor NFATc1 and its downstream proteins, thereby inhibiting osteoclast formation and function. *In vivo* studies, OA could protect against both the ovariectomy-induced bone loss model and the LPS-mediated osteolysis model. OA could be used as a potential drug against osteoporosis and osteolysis. Rhaponticin (Rh) is a natural compound isolated from the Chinese herb rhubarb. He et al. (2021) found that Rh could reduce ROS-induced oxidative stress by enhancing the activities of antioxidant enzymes such as CAT, SOD-2, and HO-1, and that RH significantly inhibited MAPK, NF-κB, and intracellular Ca^2+^ oscillation to suppress osteoclastogenesis. Alpinetin (Alp), a natural flavonoid, is the main active ingredient of Alpinia katsumadai Hayat, with significant anti-inflammatory, anti-tumor and antioxidant properties. Wei et al. (2022) found that Alp inhibited intracellular ROS levels by regulating transcription factor Nrf2-mediated antioxidant enzymes and downregulating NADPH oxidase expression, ultimately reducing the cellular activity of OCs, and also demonstrated the therapeutic effect of Alp on inflammatory bone loss through *in vitro* and *in vivo* studies.

## Conclusion

**FIGURE 2:**
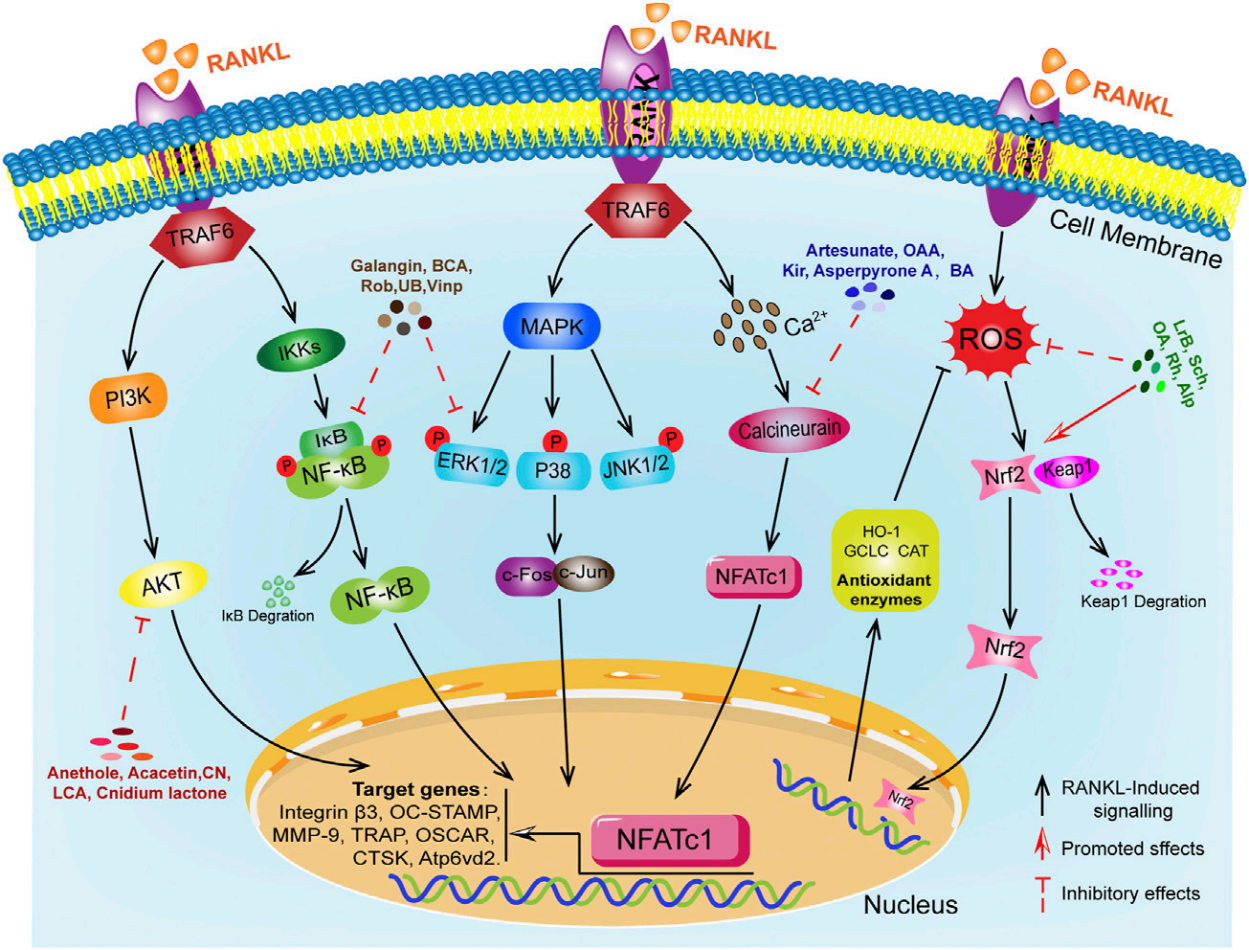
RANKL-mediated osteoclast differentiation signaling pathway and 20 natural compounds.

Abnormal osteoclast differentiation is the main pathological cause of osteoporosis. RANKL-induced downstream multiple signaling pathways are key steps in osteoclast differentiation, and abnormal function of these downstream signaling molecules can lead to disorders in osteoclast differentiation and function. This paper reviews the recent research progress in the study of signaling pathways related to RANKL-induced OCs differentiation, but the specific pathways and molecular mechanisms of OCs differentiation are currently poorly understood and require further investigation. A number of anti-osteoporosis drugs have been used in clinical practice, but their long-term use has frequently caused some adverse effects. Therefore, there is a need to increase our exploration of natural plant derivatives and natural compounds against osteoporosis. As shown in Figure 2, the biological effects of natural compounds on osteoclast differentiation and their mechanism of action on downstream signaling. Natural drugs are extremely advantageous and unique in the treatment of osteoporosis. These natural active compounds from plants show anti-bone destructive activity *in vitro* and *in vivo* by modulating multiple signaling pathways and show excellent results. This review focuses on highlighting the importance of natural drugs in the treatment of osteoclast-mediated bone destructive diseases. Natural products with osteoclasts as therapeutic targets will have great potential in the prevention and treatment of osteolytic diseases. However, current research relies on *in vitro* and *in vivo* animal studies, and no validated *in vivo* human experiments have been found. In the future, we need advanced techniques to isolate more optimized active compounds from herbal medicines for the prevention and treatment of osteolytic diseases and to further explore their exact molecular mechanisms. This will help to provide new therapeutic ideas and approaches for osteolytic diseases associated with osteoclasts.

## References

[B1] Abu-Amer Y. (2013). NF-κB signaling and bone resorption. Osteoporos. Int. 24, 2377–2386. 10.1007/s00198-013-2313-x 23468073 PMC3884829

[B2] Agidigbi T. S. Kim C. (2019). Reactive oxygen species in osteoclast differentiation and possible pharmaceutical targets of ROS-mediated osteoclast diseases. Int. J. Mol. Sci. 20, E3576. 10.3390/ijms20143576 PMC6678498 31336616

[B3] An J. Yang H. Zhang Q. Liu C. Zhao J. Zhang L. (2016). Natural products for treatment of osteoporosis: the effects and mechanisms on promoting osteoblast-mediated bone formation. Life Sci. 147, 46–58. 10.1016/j.lfs.2016.01.024 26796578

[B4] Ayub N. Faraj M. Ghatan S. Reijers J. a. A. Napoli N. Oei L. (2021). The treatment gap in osteoporosis. J. Clin. Med. 10, 3002. 10.3390/jcm10133002 34279485 PMC8268346

[B5] Bacevic M. Brkovic B. Albert A. Rompen E. Radermecker R. P. Lambert F. (2017). Does oxidative stress play a role in altered characteristics of diabetic bone? A systematic review. Calcif. Tissue Int. 101, 553–563. 10.1007/s00223-017-0327-7 29063963

[B6] Bang E. Kim D. H. Chung H. Y. (2021). Protease-activated receptor 2 induces ROS-mediated inflammation through Akt-mediated NF-κB and FoxO6 modulation during skin photoaging. Redox Biol. 44, 102022. 10.1016/j.redox.2021.102022 34082382 PMC8182111

[B7] Bone H. G. Wagman R. B. Brandi M. L. Brown J. P. Chapurlat R. Cummings S. R. (2017). 10 years of denosumab treatment in postmenopausal women with osteoporosis: results from the phase 3 randomised FREEDOM trial and open-label extension. Lancet. Diabetes Endocrinol. 5, 513–523. 10.1016/S2213-8587(17)30138-9 28546097

[B8] Boyce B. F. Yao Z. Xing L. (2010). Functions of nuclear factor kappaB in bone. Ann. N. Y. Acad. Sci. 1192, 367–375. 10.1111/j.1749-6632.2009.05315.x 20392262 PMC3013362

[B9] Boyle W. J. Simonet W. S. Lacey D. L. (2003). Osteoclast differentiation and activation. Nature 423, 337–342. 10.1038/nature01658 12748652

[B10] Chen D. Wang Q. Li Y. Sun P. Kuek V. Yuan J. (2021). Notopterol attenuates estrogen deficiency-induced osteoporosis via repressing RANKL signaling and reactive oxygen species. Front. Pharmacol. 12, 664836. 10.3389/fphar.2021.664836 34149419 PMC8210423

[B11] Chen X. Wang C. Qiu H. Yuan Y. Chen K. Cao Z. (2019). Asperpyrone A attenuates RANKL-induced osteoclast formation through inhibiting NFATc1, Ca(2+) signalling and oxidative stress. J. Cell. Mol. Med. 23, 8269–8279. 10.1111/jcmm.14700 31612613 PMC6850946

[B12] Clézardin P. Coleman R. Puppo M. Ottewell P. Bonnelye E. Paycha F. (2021). Bone metastasis: mechanisms, therapies, and biomarkers. Physiol. Rev. 101, 797–855. 10.1152/physrev.00012.2019 33356915

[B13] Compston J. E. Mcclung M. R. Leslie W. D. (2019). Osteoporosis. Lancet 393, 364–376. 10.1016/S0140-6736(18)32112-3 30696576

[B14] Cornelius C. Koverech G. Crupi R. Di Paola R. Koverech A. Lodato F. (2014). Osteoporosis and alzheimer pathology: Role of cellular stress response and hormetic redox signaling in aging and bone remodeling. Front. Pharmacol. 5, 120. 10.3389/fphar.2014.00120 24959146 PMC4050335

[B15] Crabtree G. R. Schreiber S. L. (2009). SnapShot: Ca2+-calcineurin-NFAT signaling. Cell 138, 210. 10.1016/j.cell.2009.06.026 19596245 PMC2958059

[B16] Curtis E. M. Moon R. J. Dennison E. M. Harvey N. C. Cooper C. (2016). Recent advances in the pathogenesis and treatment of osteoporosis. Clin. Med. 16, 360–364. 10.7861/clinmedicine.16-4-360 PMC5011415 27481382

[B17] Da W. Tao L. Zhu Y. (2021). The role of osteoclast energy metabolism in the occurrence and development of osteoporosis. Front. Endocrinol. 12, 675385. 10.3389/fendo.2021.675385 PMC8150001 34054735

[B18] Denicola G. M. Karreth F. A. Humpton T. J. Gopinathan A. Wei C. Frese K. (2011). Oncogene-induced Nrf2 transcription promotes ROS detoxification and tumorigenesis. Nature 475, 106–109. 10.1038/nature10189 21734707 PMC3404470

[B19] Erkhembaatar M. Gu D. R. Lee S. H. Yang Y. M. Park S. Muallem S. (2017). Lysosomal Ca(2+) signaling is essential for osteoclastogenesis and bone remodeling. J. Bone Min. Res. 32, 385–396. 10.1002/jbmr.2986 PMC9850942 27589205

[B20] Fattahi S. Amjadi-Moheb F. Tabaripour R. Ashrafi G. H. Akhavan-Niaki H. (2020). PI3K/AKT/mTOR signaling in gastric cancer: Epigenetics and beyond. Life Sci. 262, 118513. 10.1016/j.lfs.2020.118513 33011222

[B21] Favus M. J. (2010). Bisphosphonates for osteoporosis. N. Engl. J. Med. 363, 2027–2035. 10.1056/NEJMct1004903 21083387

[B22] Feng X. Teitelbaum S. L. (2013). Osteoclasts: New insights. Bone Res. 1, 11–26. 10.4248/BR201301003 26273491 PMC4472093

[B23] Go Y. M. Jones D. P. (2017). Redox theory of aging: implications for health and disease. Clin. Sci. 131, 1669–1688. 10.1042/CS20160897 PMC5773128 28667066

[B24] Han J. Yang K. An J. Jiang N. Fu S. Tang X. (2022). The role of NRF2 in bone metabolism - friend or foe? Front. Endocrinol. 13, 813057. 10.3389/fendo.2022.813057 PMC8906930 35282459

[B25] He J. Chen K. Deng T. Xie J. Zhong K. Yuan J. (2021). Inhibitory effects of rhaponticin on osteoclast formation and resorption by targeting RANKL-induced NFATc1 and ROS activity. Front. Pharmacol. 12, 645140. 10.3389/fphar.2021.645140 34630071 PMC8495440

[B26] Hinz N. Jücker M. (2021). AKT in bone metastasis of solid tumors: a comprehensive review. Cancers (Basel) 13, 2287. 10.3390/cancers13102287 34064589 PMC8151478

[B27] Hirata N. Ichimaru R. Tominari T. Matsumoto C. Watanabe K. Taniguchi K. (2019). Beta-cryptoxanthin inhibits lipopolysaccharide-induced osteoclast differentiation and bone resorption via the suppression of inhibitor of NF-κB kinase activity. Nutrients 11, E368. 10.3390/nu11020368 PMC6412436 30744180

[B28] Hong G. Chen Z. Han X. Zhou L. Pang F. Wu R. (2021). A novel RANKL-targeted flavonoid glycoside prevents osteoporosis through inhibiting NFATc1 and reactive oxygen species. Clin. Transl. Med. 11, e392. 10.1002/ctm2.392 34047464 PMC8140192

[B29] Hu Y. Li X. Zhi X. Cong W. Huang B. Chen H. (2021). RANKL from bone marrow adipose lineage cells promotes osteoclast formation and bone loss. EMBO Rep. 22, e52481. 10.15252/embr.202152481 34121311 PMC8406405

[B30] Irelli A. Sirufo M. M. Scipioni T. De Pietro F. Pancotti A. Ginaldi L. (2019). mTOR links tumor immunity and bone metabolism: What are the clinical implications? Int. J. Mol. Sci. 20, E5841. 10.3390/ijms20235841 PMC6928935 31766386

[B31] Jeong D. H. Kwak S. C. Lee M. S. Yoon K. H. Kim J. Y. Lee C. H. (2020). Betulinic acid inhibits RANKL-induced osteoclastogenesis via attenuating Akt, NF-κB, and PLCγ2-Ca(2+) signaling and prevents inflammatory bone loss. J. Nat. Prod. 83, 1174–1182. 10.1021/acs.jnatprod.9b01212 32237724

[B32] Jimi E. Takakura N. Hiura F. Nakamura I. Hirata-Tsuchiya S. (2019). The role of NF-κB in physiological bone development and inflammatory bone diseases: Is NF-κB inhibition "killing two birds with one stone? Cells 8, E1636. 10.3390/cells8121636 PMC6952937 31847314

[B33] Jo Y. J. Lee H. I. Kim N. Hwang D. Lee J. Lee G. R. (2021). Cinchonine inhibits osteoclast differentiation by regulating TAK1 and AKT, and promotes osteogenesis. J. Cell. Physiol. 236, 1854–1865. 10.1002/jcp.29968 32700766

[B34] Johnston C. B. Dagar M. (2020). Osteoporosis in older adults. Med. Clin. North Am. 104, 873–884. 10.1016/j.mcna.2020.06.004 32773051

[B35] Kajiya H. (2012). Calcium signaling in osteoclast differentiation and bone resorption. Adv. Exp. Med. Biol. 740, 917–932. 10.1007/978-94-007-2888-2_41 22453976

[B36] Kang J. Y. Kang N. Yang Y. M. Hong J. H. Shin D. M. (2020). The role of Ca(2+)-NFATc1 signaling and its modulation on osteoclastogenesis. Int. J. Mol. Sci. 21, E3646. 10.3390/ijms21103646 PMC7279283 32455661

[B37] Kawamura N. Kugimiya F. Oshima Y. Ohba S. Ikeda T. Saito T. (2007). Akt1 in osteoblasts and osteoclasts controls bone remodeling. PLoS One 2, e1058. 10.1371/journal.pone.0001058 17957242 PMC2020440

[B38] Kim J. M. Lin C. Stavre Z. Greenblatt M. B. Shim J. H. (2020). Osteoblast-osteoclast communication and bone homeostasis. Cells 9, E2073. 10.3390/cells9092073 PMC7564526 32927921

[B39] Kim J. Y. Cheon Y. H. Oh H. M. Rho M. C. Erkhembaatar M. Kim M. S. (2014). Oleanolic acid acetate inhibits osteoclast differentiation by downregulating PLCγ2-Ca(2+)-NFATc1 signaling, and suppresses bone loss in mice. Bone 60, 104–111. 10.1016/j.bone.2013.12.013 24361669

[B40] Kodama J. Kaito T. (2020). Osteoclast multinucleation: Review of current literature. Int. J. Mol. Sci. 21, E5685. 10.3390/ijms21165685 PMC7461040 32784443

[B41] Kong L. Wang B. Yang X. He B. Hao D. Yan L. (2020). Integrin-associated molecules and signalling cross talking in osteoclast cytoskeleton regulation. J. Cell. Mol. Med. 24, 3271–3281. 10.1111/jcmm.15052 32045092 PMC7131929

[B42] Kraenzlin M. E. Meier C. (2011). Parathyroid hormone analogues in the treatment of osteoporosis. Nat. Rev. Endocrinol. 7, 647–656. 10.1038/nrendo.2011.108 21750510

[B43] Lee K. Chung Y. H. Ahn H. Kim H. Rho J. Jeong D. (2016). Selective regulation of MAPK signaling mediates RANKL-dependent osteoclast differentiation. Int. J. Biol. Sci. 12, 235–245. 10.7150/ijbs.13814 26884720 PMC4737679

[B44] Lee K. Seo I. Choi M. H. Jeong D. (2018). Roles of mitogen-activated protein kinases in osteoclast biology. Int. J. Mol. Sci. 19, E3004. 10.3390/ijms19103004 PMC6213329 30275408

[B45] Li J. Sarosi I. Yan X. Q. Morony S. Capparelli C. Tan H. L. (2000). RANK is the intrinsic hematopoietic cell surface receptor that controls osteoclastogenesis and regulation of bone mass and calcium metabolism. Proc. Natl. Acad. Sci. U. S. A. 97, 1566–1571. 10.1073/pnas.97.4.1566 10677500 PMC26475

[B46] Li X. Jiang J. Yang Z. Jin S. Lu X. Qian Y. (2021a). Galangin suppresses RANKL-induced osteoclastogenesis via inhibiting MAPK and NF-κB signalling pathways. J. Cell. Mol. Med. 25, 4988–5000. 10.1111/jcmm.16430 33939240 PMC8178255

[B47] Li X. Li B. Shi Y. Wang C. Ye L. (2021b). Targeting reactive oxygen species in stem cells for bone therapy. Drug Discov. Today 26, 1226–1244. 10.1016/j.drudis.2021.03.002 33684524

[B48] Liang J. Y. Wu W. L. Chen Y. X. Liu H. (2019). The efficacy and potential mechanism of cnidium lactone to inhibit osteoclast differentiation. Artif. Cells Nanomed. Biotechnol. 47, 3087–3093. 10.1080/21691401.2019.1637881 31343277

[B49] Liao S. Feng W. Liu Y. Wang Z. Ding X. Song F. (2021). Inhibitory effects of biochanin A on titanium particle-induced osteoclast activation and inflammatory bone resorption via NF-κB and MAPK pathways. J. Cell. Physiol. 236, 1432–1444. 10.1002/jcp.29948 32853427

[B50] Liel Y. (2018). Teriparatide vs risedronate for osteoporosis. Lancet 391, 1895. 10.1016/S0140-6736(18)30754-2 29781441

[B51] Lin X. Xu F. Zhang K. W. Qiu W. X. Zhang H. Hao Q. (2022). Acacetin prevents bone loss by disrupting osteoclast formation and promoting type H vessel formation in ovariectomy-induced osteoporosis. Front. Cell Dev. Biol. 10, 796227. 10.3389/fcell.2022.796227 35517504 PMC9062130

[B52] Liu W. Le C. C. Wang D. Ran D. Wang Y. Zhao H. (2020). Ca(2+)/CaM/CaMK signaling is involved in cadmium-induced osteoclast differentiation. Toxicology 441, 152520. 10.1016/j.tox.2020.152520 32522522

[B53] Liu Y. Wang C. Wang G. Sun Y. Deng Z. Chen L. (2019). Loureirin B suppresses RANKL-induced osteoclastogenesis and ovariectomized osteoporosis via attenuating NFATc1 and ROS activities. Theranostics 9, 4648–4662. 10.7150/thno.35414 31367247 PMC6643439

[B54] Lorenzo J. (2017). The many ways of osteoclast activation. J. Clin. Invest. 127, 2530–2532. 10.1172/JCI94606 28530641 PMC5490742

[B55] Lu L. Rao L. Jia H. Chen J. Lu X. Yang G. (2017). Baicalin positively regulates osteoclast function by activating MAPK/Mitf signalling. J. Cell. Mol. Med. 21, 1361–1372. 10.1111/jcmm.13066 28158928 PMC5487921

[B56] Madreiter-Sokolowski C. T. Thomas C. Ristow M. (2020). Interrelation between ROS and Ca(2+) in aging and age-related diseases. Redox Biol. 36, 101678. 10.1016/j.redox.2020.101678 32810740 PMC7451758

[B57] Mandal C. C. Ghosh Choudhury G. Ghosh-Choudhury N. (2009). Phosphatidylinositol 3 kinase/Akt signal relay cooperates with smad in bone morphogenetic protein-2-induced colony stimulating factor-1 (CSF-1) expression and osteoclast differentiation. Endocrinology 150, 4989–4998. 10.1210/en.2009-0026 19819979 PMC2775973

[B58] Mcdonald M. M. Khoo W. H. Ng P. Y. Xiao Y. Zamerli J. Thatcher P. (2021). Osteoclasts recycle via osteomorphs during RANKL-stimulated bone resorption. Cell 184, 1940–1347. 10.1016/j.cell.2021.03.010 33798441 PMC8024244

[B59] Miyazaki T. Sanjay A. Neff L. Tanaka S. Horne W. C. Baron R. (2004). Src kinase activity is essential for osteoclast function. J. Biol. Chem. 279, 17660–17666. 10.1074/jbc.M311032200 14739300

[B60] Moon J. B. Kim J. H. Kim K. Youn B. U. Ko A. Lee S. Y. (2012). Akt induces osteoclast differentiation through regulating the GSK3β/NFATc1 signaling cascade. J. Immunol. 188, 163–169. 10.4049/jimmunol.1101254 22131333

[B61] Morgan M. J. Liu Z. G. (2011). Crosstalk of reactive oxygen species and NF-κB signaling. Cell Res. 21, 103–115. 10.1038/cr.2010.178 21187859 PMC3193400

[B62] Nakamura I. Takahashi N. Sasaki T. Tanaka S. Udagawa N. Murakami H. (1995). Wortmannin, a specific inhibitor of phosphatidylinositol-3 kinase, blocks osteoclastic bone resorption. FEBS Lett. 361, 79–84. 10.1016/0014-5793(95)00153-z 7890044

[B63] Ni S. Qian Z. Yuan Y. Li D. Zhong Z. Ghorbani F. (2020). Schisandrin A restrains osteoclastogenesis by inhibiting reactive oxygen species and activating Nrf2 signalling. Cell Prolif. 53, e12882. 10.1111/cpr.12882 32871020 PMC7574870

[B64] Novack D. V. (2011). Role of NF-κB in the skeleton. Cell Res. 21, 169–182. 10.1038/cr.2010.159 21079651 PMC3193402

[B65] Oikawa A. Kobayashi M. Okamatsu Y. Shinki T. Kamijo R. Yamamoto M. (2007). Mitogen-activated protein kinases mediate interleukin-1beta-induced receptor activator of nuclear factor-kappaB ligand expression in human periodontal ligament cells. J. Periodontal Res. 42, 367–376. 10.1111/j.1600-0765.2006.00959.x 17559635

[B66] Okada H. Okabe K. Tanaka S. (2020). Finely-tuned calcium oscillations in osteoclast differentiation and bone resorption. Int. J. Mol. Sci. 22, E180. 10.3390/ijms22010180 PMC7794828 33375370

[B67] Oryan A. Sahvieh S. (2021). Effects of bisphosphonates on osteoporosis: Focus on zoledronate. Life Sci. 264, 118681. 10.1016/j.lfs.2020.118681 33129881

[B68] Pang K. L. Low N. Y. Chin K. Y. (2020). A review on the role of denosumab in fracture prevention. Drug Des. Devel. Ther. 14, 4029–4051. 10.2147/DDDT.S270829 PMC7534845 33061307

[B69] Parveen B. Parveen A. Vohora D. (2019). Biomarkers of osteoporosis: an update. Endocr. Metab. Immune Disord. Drug Targets 19, 895–912. 10.2174/1871530319666190204165207 30727928

[B70] Polyzos S. A. Makras P. Tournis S. Anastasilakis A. D. (2019). Off-label uses of denosumab in metabolic bone diseases. Bone 129, 115048. 10.1016/j.bone.2019.115048 31454537

[B71] Qu H. Zhang Y. He R. Lin N. Wang C. (2021). Anethole inhibits RANKL-induced osteoclastogenesis by downregulating ERK/AKT signaling and prevents ovariectomy-induced bone loss *in vivo* . Int. Immunopharmacol. 100, 108113. 10.1016/j.intimp.2021.108113 34530203

[B72] Qu Z. An H. Feng M. Huang W. Wang D. Zhang Z. (2022). Urolithin B suppresses osteoclastogenesis via inhibiting RANKL-induced signalling pathways and attenuating ROS activities. J. Cell. Mol. Med. 26, 4428–4439. 10.1111/jcmm.17467 35781786 PMC9357644

[B73] Rachner T. D. Khosla S. Hofbauer L. C. (2011). Osteoporosis: now and the future. Lancet 377, 1276–1287. 10.1016/S0140-6736(10)62349-5 21450337 PMC3555696

[B74] Reid I. R. Billington E. O. (2022). Drug therapy for osteoporosis in older adults. Lancet 399, 1080–1092. 10.1016/S0140-6736(21)02646-5 35279261

[B75] Ren R. Guo J. Chen Y. Zhang Y. Chen L. Xiong W. (2021). The role of Ca(2+) /Calcineurin/NFAT signalling pathway in osteoblastogenesis. Cell Prolif. 54, e13122. 10.1111/cpr.13122 34523757 PMC8560623

[B76] Rozenberg S. Al-Daghri N. Aubertin-Leheudre M. Brandi M. L. Cano A. Collins P. (2020). Is there a role for menopausal hormone therapy in the management of postmenopausal osteoporosis? Osteoporos. Int. 31, 2271–2286. 10.1007/s00198-020-05497-8 32642851 PMC7661391

[B77] Sánchez-De-Diego C. Pedrazza L. Pimenta-Lopes C. Martinez-Martinez A. Dahdah N. Valer J. A. (2021). NRF2 function in osteocytes is required for bone homeostasis and drives osteocytic gene expression. Redox Biol. 40, 101845. 10.1016/j.redox.2020.101845 33373776 PMC7773566

[B78] Smith M. V. Lee M. J. Islam A. S. Rohrer J. L. Goldberg V. M. Beidelschies M. A. (2007). Inhibition of the PI3K-Akt signaling pathway reduces tumor necrosis factor-alpha production in response to titanium particles *in vitro* . J. Bone Jt. Surg. Am. 89, 1019–1027. 10.2106/JBJS.F.00615 17473139

[B79] Takeshita S. Kaji K. Kudo A. (2000). Identification and characterization of the new osteoclast progenitor with macrophage phenotypes being able to differentiate into mature osteoclasts. J. Bone Min. Res. 15, 1477–1488. 10.1359/jbmr.2000.15.8.1477 10934646

[B80] Tu W. Wang H. Li S. Liu Q. Sha H. (2019). The anti-inflammatory and anti-oxidant mechanisms of the keap1/nrf2/ARE signaling pathway in chronic diseases. Aging Dis. 10, 637–651. 10.14336/AD.2018.0513 31165007 PMC6538222

[B81] Udagawa N. Koide M. Nakamura M. Nakamichi Y. Yamashita T. Uehara S. (2021). Osteoclast differentiation by RANKL and OPG signaling pathways. J. Bone Min. Metab. 39, 19–26. 10.1007/s00774-020-01162-6 33079279

[B82] Verron E. Bouler J. M. (2014). Is bisphosphonate therapy compromised by the emergence of adverse bone disorders? Drug Discov. Today 19, 312–319. 10.1016/j.drudis.2013.08.010 23974069

[B83] Wang D. Liu Y. Tang D. Wei S. Sun J. Ruan L. (2021). Induction of PI3K/Akt-Mediated apoptosis in osteoclasts is a key approach for buxue tongluo pills to treat osteonecrosis of the femoral head. Front. Pharmacol. 12, 729909. 10.3389/fphar.2021.729909 34912214 PMC8667870

[B84] Wang Y. Liu L. Qu Z. Wang D. Huang W. Kong L. (2022). Tanshinone ameliorates glucocorticoid-induced bone loss via activation of AKT1 signaling pathway. Front. Cell Dev. Biol. 10, 878433. 10.3389/fcell.2022.878433 35419360 PMC8995529

[B85] Wang Z. Bao H. W. Xu Y. J. (2020). Cnidium lactone prevents bone loss in an ovariectomized rat model through the estrogen-α/BMP-2/Smad signaling pathway. J. Gene Med. 22, e3198. 10.1002/jgm.3198 32267602

[B86] Wei L. Chen W. Huang L. Wang H. Su Y. Liang J. (2022). Alpinetin ameliorates bone loss in LPS-induced inflammation osteolysis via ROS mediated P38/PI3K signaling pathway. Pharmacol. Res. 184, 106400. 10.1016/j.phrs.2022.106400 35988868

[B87] Wysowski D. K. (2009). Reports of esophageal cancer with oral bisphosphonate use. N. Engl. J. Med. 360, 89–90. 10.1056/NEJMc0808738 19118315

[B88] Xian Y. Su Y. Liang J. Long F. Feng X. Xiao Y. (2021). Oroxylin A reduces osteoclast formation and bone resorption via suppressing RANKL-induced ROS and NFATc1 activation. Biochem. Pharmacol. 193, 114761. 10.1016/j.bcp.2021.114761 34492273

[B89] Xiao L. Zhong M. Huang Y. Zhu J. Tang W. Li D. (2020). Puerarin alleviates osteoporosis in the ovariectomy-induced mice by suppressing osteoclastogenesis via inhibition of TRAF6/ROS-dependent MAPK/NF-κB signaling pathways. Aging (Albany NY) 12, 21706–21729. 10.18632/aging.103976 33176281 PMC7695364

[B90] Yu L. Jia D. Feng K. Sun X. Xu W. Ding L. (2020). A natural compound (LCA) isolated from Litsea cubeba inhibits RANKL-induced osteoclast differentiation by suppressing Akt and MAPK pathways in mouse bone marrow macrophages. J. Ethnopharmacol. 257, 112873. 10.1016/j.jep.2020.112873 32298753

[B91] Zeng X. Z. Zhang Y. Y. Yang Q. Wang S. Zou B. H. Tan Y. H. (2020). Artesunate attenuates LPS-induced osteoclastogenesis by suppressing TLR4/TRAF6 and PLCγ1-Ca(2+)-NFATc1 signaling pathway. Acta Pharmacol. Sin. 41, 229–236. 10.1038/s41401-019-0289-6 31431733 PMC7468527

[B92] Zhang H. J. Liao H. Y. Bai D. Y. Wang Z. Q. Xie X. W. (2021). MAPK /ERK signaling pathway: A potential target for the treatment of intervertebral disc degeneration. Biomed. Pharmacother. 143, 112170. 10.1016/j.biopha.2021.112170 34536759

[B93] Zhang J. Wang X. Vikash V. Ye Q. Wu D. Liu Y. (2016). ROS and ROS-mediated cellular signaling. Oxid. Med. Cell. Longev. 2016, 4350965. 10.1155/2016/4350965 26998193 PMC4779832

[B94] Zhu C. Shen S. Zhang S. Huang M. Zhang L. Chen X. (2022). Autophagy in bone remodeling: A regulator of oxidative stress. Front. Endocrinol. 13, 898634. 10.3389/fendo.2022.898634 PMC9279723 35846332

[B95] Zhu M. Liu H. Sun K. Liu J. Mou Y. Qi D. (2020). Vinpocetine inhibits RANKL-induced osteoclastogenesis and attenuates ovariectomy-induced bone loss. Biomed. Pharmacother. 123, 109769. 10.1016/j.biopha.2019.109769 31846839

[B96] Zou B. Zheng J. Deng W. Tan Y. Jie L. Qu Y. (2021). Kirenol inhibits RANKL-induced osteoclastogenesis and prevents ovariectomized-induced osteoporosis via suppressing the Ca(2+)-NFATc1 and Cav-1 signaling pathways. Phytomedicine 80, 153377. 10.1016/j.phymed.2020.153377 33126167

